# Low Frequency Electroacupuncture Alleviated Spinal Nerve Ligation Induced Mechanical Allodynia by Inhibiting TRPV1 Upregulation in Ipsilateral Undamaged Dorsal Root Ganglia in Rats

**DOI:** 10.1155/2013/170910

**Published:** 2013-07-10

**Authors:** Yong-Liang Jiang, Xiao-Hu Yin, Ya-Fang Shen, Xiao-Fen He, Jian-Qiao Fang

**Affiliations:** Department of Neurobiology and Acupuncture Research, the Third Clinical Medical College, Zhejiang Chinese Medical University, Hangzhou 310053, China

## Abstract

Neuropathic pain is an intractable problem in clinical practice. Accumulating evidence shows that electroacupuncture (EA) with low frequency can effectively relieve neuropathic pain. Transient receptor potential vanilloid type 1 (TRPV1) plays a key role in neuropathic pain. The study aimed to investigate whether neuropathic pain relieved by EA administration correlates with TRPV1 inhibition. Neuropathic pain was induced by right L5 spinal nerve ligation (SNL) in rats. 2 Hz EA stimulation was administered. SNL induced mechanical allodynia in ipsilateral hind paw. SNL caused a significant reduction of TRPV1 expression in ipsilateral L5 dorsal root ganglia (DRG), but a significant up-regulation in ipsilateral L4 and L6 DRGs. Calcitonin gene-related peptide (CGRP) change was consistent with that of TRPV1. EA alleviated mechanical allodynia, and inhibited TRPV1 and CGRP overexpressions in ipsilateral L4 and L6 DRGs. SNL did not decrease pain threshold of contralateral hind paw, and TRPV1 expression was not changed in contralateral L5 DRG. 0.001, 0.01 mg/kg TRPV1 agonist 6′-IRTX fully blocked EA analgesia in ipsilateral hind paw. 0.01 mg/kg 6′-IRTX also significantly decreased pain threshold of contralateral paw. These results indicated that inhibition of TRPV1 up-regulation in ipsilateral adjacent undamaged DRGs contributed to low frequency EA analgesia for mechanical allodynia induced by spinal nerve ligation.

## 1. Introduction

Neuropathic pain such as painful diabetic neuropathy, postherpetic neuralgia, trigeminal neuralgia, and poststroke pain is an intractable problem in clinical practice. It results from lesions or disease affecting the somatosensory nervous system either in the periphery or centrally and is characterized by spontaneous pain, allodynia, and hyperalgisea [1]. Up to now, the medication treatment of neuropathic pain is still unsatisfactory [2]. 

Abnormalities of channels or receptors in sensory nociceptors and dorsal root ganglia (DRG) are closely related to neuropathic pain [2, 3]. Transient receptor potential vanilloid type 1 (TRPV1), an important signal integrator in sensory nociceptors, plays a key role in neuropathic pain [4]. TRPV1 up-regulation contributes to mechanical allodynia and thermal hyperalgisea caused by various nerve injuries, while its antagonists can reverse the allodynia and hyperalgisea [5–7]. Furthermore, TRPV1 is coexpressed with various neuropeptides including calcitonin gene-related peptide (CGRP) in sensory ganglia and small sensory C and A*δ* fibers [8, 9]. Activation of TRPV1 promotes CGRP release from nerve terminals [10, 11], which further aggravates neuropathic pain [12, 13].

Electroacupuncture (EA), a commonly used acupuncture method applying a pulsating electrical current to acupuncture needles for acupoints stimulation, has been widely adopted for pain relief for decades [14]. Accumulating evidence shows that EA is effective in relieving neuropathic pain under multiple conditions like painful diabetic neuropathy, nerve ligation, injury, and chronic constriction [15–19]. A former study showed that EA with low frequency reduced nociceptive response and normalized TRPV1 abnormalities in hind paw skin and DRG induced by nerve growth factor injection in rats [20]. However, little is known about the effect of EA with low frequency on TRPV1 under neuropathic pain condition, and whether this effect contributes to its alleviation of neuropathic pain.

For this sake, the rat neuropathic pain model induced by right L5 spinal nerve ligation (SNL) was used in the current study. The effects of EA with low frequency on mechanical allodynia and the expressions of TRPV1 and CGRP in ipsilateral L4–6 and contralateral L5 DRGs were systematically examined. A block study using TRPV1 agonist was also performed to further ascertain the involvement of TRPV1 inhibition in neuropathic pain relief by low frequency EA.

## 2. Materials and Methods

### 2.1. Animal Preparation

Male Sprague-Dawley rats (180–200 g body weight) were obtained from SLAC Laboratory Animal Co. Ltd., Shanghai, China. Rats were housed in temperature-controlled animal cages (25 ± 1°C) under a 12 h light and 12 h dark cycle, with free access to food and water. All animals were treated in accordance with the regulations of the State Science and Technology Commission for the care and use of laboratory animals (State Science and Technology Commission Order no. 2, 1988).

### 2.2. Experimental Design

Two experiments were conducted: (1) effects of low frequency EA on allodynia-like behavior and profiles of TRPV1 and CGRP in DRGs of rats with SNL-induced neuropathic pain and (2) effects of TRPV1 receptor agonist 6′-iodoresiniferatoxin (6′-IRTX) on EA action. In experiment 1, rats were randomly divided into the following groups (*n* = 8 per group): normal, sham, SNL, and SNL + 2 Hz EA. In experiment 2, SNL-induced neuropathic pain rats were randomly divided into SNL + 2 Hz EA + vehicle, SNL + 2 Hz EA + 0.001 mg/kg 6′-IRTX, and SNL + 2 Hz EA + 0.01 mg/kg 6′-IRTX groups (*n* = 8 per group).

### 2.3. Spinal Nerve Ligation

L5 SNL was performed as previously described [21]. Briefly, rats were anesthetized with chloral hydrate (300 mg/kg, i.p.) and placed under a microsurgical apparatus in a prone position. A midline incision was made at the L3–S2 level, and the dorsal vertebral column from L4 to S2 was exposed. The right L6 transverse process was carefully removed, and then the right L5 spinal nerve was carefully isolated and then tightly ligated with a 6-0 silk thread. Sham-operated animals were subjected to the same surgical procedure except that the isolated L5 spinal nerve was not ligated.

### 2.4. EA Treatment

Acupuncture needles of 0.25 mm in diameter were inserted approximately 5 mm deep into the ipsilateral acupoints Zusanli (ST36, 5 mm lateral to the anterior tubercule of the tibia) and Kunlun (BL60, at the ankle joint level and between the tip of the external malleolus and tendo calcaneus). The ends of the needles were attached to a pair of electrodes from an electrical stimulator (LH-202H, Huawei Co. Ltd., China). EA (2 Hz, 2 mA, 0.4 ms pulse width) was administered for 30 minutes once every other day from day 3 to day 15 after surgery. Since the analgesic effects of the two acupoints are well documented [10, 21], we did not carry out sham acupuncture for control. Animals were awake and calmed by placing the heads in black hoods with no physical restraint during EA treatment. Rats were subject to the same calming procedure in normal, sham, and SNL groups.

### 2.5. Drug Delivery

In experiment 2, 1 mg/mL 6′-IRTX (Sigma, USA) solution was made by dissolving 6′-IRTX in a 95% ethanol mixture which was used as the vehicle control. 6′-IRTX was intraperitoneally injected (0.001 mg/kg, 0.01 mg/kg, resp.) in a volume of 1 mL by adding saline 10 minutes before EA treatment on day 15. 

### 2.6. Behavioral Testing

All tests were performed by an experimenter blinded to the treatment groups. After habituation, paw withdrawal threshold to a von Frey-like filament was measured to assess mechanical allodynia using a Dynamic Plantar Aesthesiometer (Ugo Basile, Italy) on day 0 (base), 1, 3, and 30 min after EA treatment on day 3, 7, 11, and 15 after surgery. The steel rod was pushed against the hind paw with linear ascending force (0–50 g) until a strong and immediate withdrawal occurred. Paw withdrawal threshold (PWT) was determined as the mean of three consecutive tests with intervals of 30 sec.

### 2.7. Immunofluorescence

Rats were deeply anesthetized by an intraperitoneal injection of 10% (w/v) chloral hydrate (3.5 mL/kg) and transcardially perfused with 150 mL cold sterilized saline followed by 500 mL cold, fresh 4% (w/v) paraformaldehyde in 0.01 M phosphate-buffered saline (PBS, pH 7.4). Contralateral L5 and ipsilateral L4, L5, and L6 DRGs were harvested, postfixed in the same fixatives for 2 h, and then consecutively immersed in 15% (w/v) and 30% (w/v) sucrose solution overnight at 4°C. DRGs were embedded in OCT (Bayer Corp., Elkhart, IN), frozen, and then cut in 10 *μ*m sections. Sections were mounted on glass slides, rinsed in PBS (pH 7.4), and blocked for 1 h at 37°C in 0.01 M PBS containing 5% (v/v) normal donkey serum and 0.3% (v/v) Triton X-100. Sections were incubated overnight at 4°C with a primary antibody (sheep anti-rat TRPV1, 1:4000; Abcam, USA) dissolved in PBS containing 0.3% (v/v) Triton X-100 and 5% (v/v) donkey serum. After being washed in PBS, sections were incubated for 1 h at 37°C with Alexa Fluor 594-conjugated donkey anti-sheep IgG (1:400; Jackson Immunoresearch, USA). After immunostaining, sections were rinsed in PBS and cover-slipped with 50% (v/v) glycerol and 2.5% (w/v) triethylenediamine (antifading agent) in 0.05 M PBS. Images were obtained using a fluorescence microscope (Olympus IX71; Olympus, Japan) equipped with Image-Pro Insight 8.0 software (Media Cybernetics, USA). 

TRPV1-immunoreactive (IR) analysis was performed as previously described [22, 23]. The observer was blinded for the treatment groups. DRG neurons with a clear nuclear profile were counted using Image-Pro Plus 6.0 (Media Cybernetics, USA). TRPV1-IR ratio was calculated by dividing the number of TRPV1-IR neurons by the total number of neurons in each section. The mean ratio was determined from three every tenth DRG sections for each DRG.

### 2.8. Western Blotting

Rats were deeply anesthetized by an intraperitoneal injection of 10% (w/v) chloral hydrate (3.5 mL/kg). Contralateral L5 and ipsilateral L4, L5, and L6 DRGs were harvested, sonicated on ice in RIPA Lysis Buffer (Beyotime, China) with an addition of protease inhibitor cocktail (Sangon Biotech, China), and centrifuged at 10,000x rpm for 10 min at 4°C, and then the supernatants were collected. Protein concentrations were determined by the bicinchoninic acid method. Protein extracts of animals in each group were equally pooled according to their concentrations. Samples were mixed with an equal volume of 2 × sample loading buffer and denatured by boiling at 100°C for 5 min. Proteins (30 *μ*g/lane) were separated by an 8% SDS-PAGE gel for TRPV1 and 15% SDS-PAGE gel for CGRP and then transferred to 0.45 *μ*m and 0.22 *μ*m PVDF membranes, respectively (0.45 *μ*m, Millipore, USA; 0.22 *μ*m, Bio-Rad, USA). After being blocked in 0.01 M TBS with 0.1% Tween 20 and 5% dehydrated skim milk, the membranes were incubated overnight at 4°C with sheep anti-rat TRPV1 (1:2000; Abcam, USA), rabbit anti-rat CGRP (1:2000; Abcam, USA), or horseradish peroxidase-conjugated mouse anti-rat *β*-actin (1:10,000; Kangcheng, China). After being washed, the membrane for *β*-actin was visualized by chemiluminescence (ECL Plus; Amersham), while the membranes for TRPV1 and CGRP were incubated with the species-specific secondary antibodies for 2 h at room temperature and then washed and visualized by chemiluminescence (ECL Plus; Amersham, USA). Bands were detected by an Image Quant LAS 4000 system (Fujifilm, Japan) with Image Quant TL 7.0 software (GE Healthcare, USA). Three independent experiments were carried out for western blotting analysis. Target protein levels were normalized against *β*-actin levels and then expressed as relative fold changes compared to the normal control group [24, 25]. 

### 2.9. Statistical Analysis

All data were expressed as mean ± SEM. Statistical analysis was performed by one-way analysis of variance (ANOVA) followed by post hoc test of the least significant difference (LSD) for multiple comparisons. *P* < 0.05 was set as the level of statistical significance.

## 3. Results

### 3.1. EA Alleviated Mechanical Allodynia Induced by SNL

**Figure 1:**
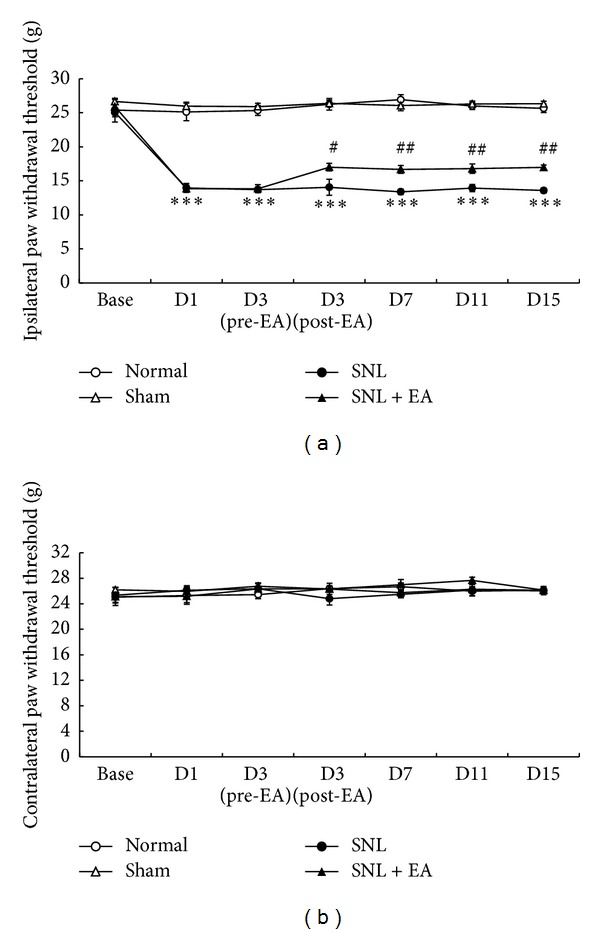
Effects of 2 Hz EA on bilateral paw withdrawal thresholds of rats subject to right L5 spinal nerve ligation. Data are presented as mean ± SEM, *n* = 8 per group. ***P < 0.001, SNL group versus normal group; ^#^ *P* < 0.05, ^##^ *P* < 0.01, SNL + EA group versus SNL group.

We first determined bilateral PWTs of rats to assess the extent of mechanical allodynia induced by SNL. Rats subjected to L5 SNL surgery developed mechanical allodynia in ipsilateral hind paw, as shown by a drastic reduction of ipsilateral PWLs on day 1, persisted to day 15 through the whole observation period (Figure 1(a)). 2 Hz EA significantly increased ipsilateral PWLs of rats subjected to SNL from day 3 after treatment, compared to the PWLs of SNL-controlled rats (*P* < 0.05, *P* < 0.01). Sham operation had no significant effect on ipsilateral PWLs during the whole period. Moreover, there was no significant difference in rats' contralateral PWTs among normal, sham-operated, SNL, and SNL + EA groups (Figure 1(b)).

### 3.2. EA Inhibited Ipsilateral Undamaged DRG TRPV1 Upregulation Resulted from SNL

**Figure 2:**
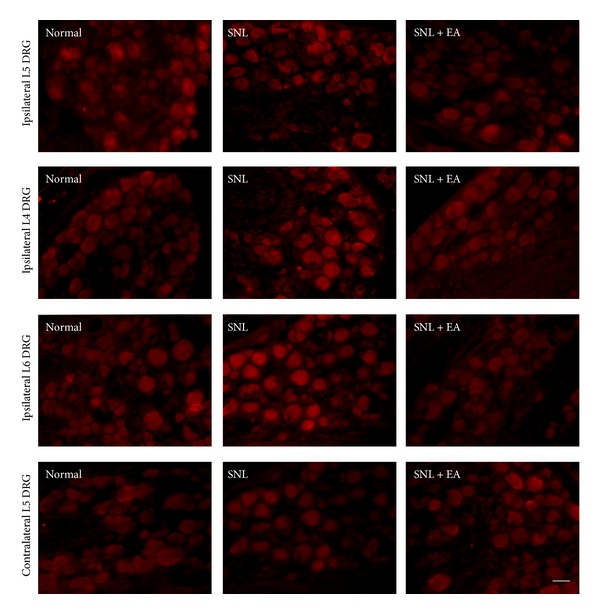
Immunofluorescence of TRPV1 expressions in ipsilateral L5, L4, and L6 and contralateral L5 DRGs in normal, SNL, and SNL + EA groups. TRPV1-IR is mainly seen in small-to-medium DRG neurons (20–50 *μ*m). Scale bar, 50 *μ*m for all.

To determine the contribution of TRPV1 to the mechanical allodynia resulted from SNL, we performed immunofluorescence study and western blotting analysis to test TRPV1 expressions and levels in ipsilateral L4, L5, and L6 and contralateral L5 DRGs of rats. Immunofluorescence showed that TRPV1 positive neurons were mainly small-to-medium DRG cells (20–50 *μ*m; Figure 2). TRPV1 expression in ipsilateral L5 DRG in SNL group (7.0 ± 1.0%) was significantly reduced as compared to that in normal group (14.7 ± 2.8%, *P* < 0.01). No significant difference of TRPV1 expression in ipsilateral L5 DRG was observed between SNL and SNL + EA groups. TRPV1 expressions in ipsilateral L4 and L6 DRGs in SNL group (18.1 ± 1.8%, 15.3 ± 1.0%) were significantly increased as compared to those in normal group (10.2 ± 1.3%, 9.4 ± 0.6%; *P* < 0.001, *P* < 0.001, resp.). The increases were inhibited by 2 Hz EA (7.3 ± 0.9%, 5.5 ± 0.7%; *P* < 0.001, *P* < 0.001, resp., compared to SNL group). Besides, there was no significant difference in TRPV1 expressions in rats' contralateral L5 DRG among normal, SNL, and SNL + EA groups.

**Figure 3:**
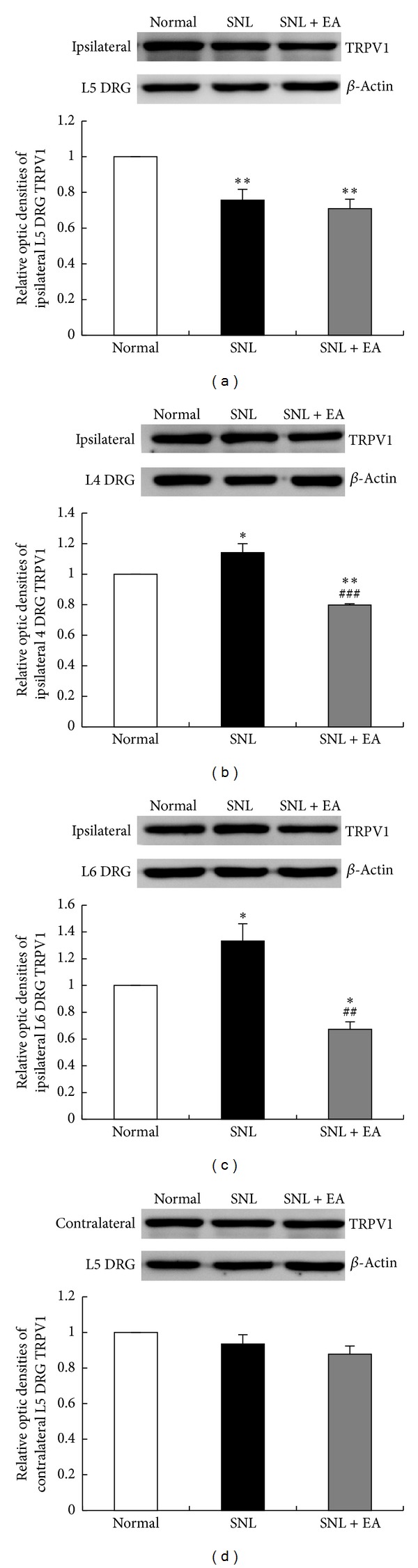
Western blotting analysis of TRPV1 levels in ipsilateral L5, L4, and L6 and contralateral L5 DRGs in normal, SNL, and SNL + EA groups. Results were expressed as relative fold changes as compared to normal group after normalization to *β*-actin. Data are presented as mean ± SEM of three independent experiments. *P < 0.05, **P < 0.01, SNL group or SNL + EA group versus normal group; ^##^ *P* < 0.01, ^###^ *P* < 0.001, SNL + EA group versus SNL group.

The changes of TRPV1 protein levels as revealed by western blotting analysis were consistent with the changes of TRPV1 expressions. TRPV1 protein level in ipsilateral L5 DRG of rats in SNL group was significantly decreased as compared to that in normal group (*P* < 0.01), and no significant difference was found between SNL and SNL + EA groups (Figure 3(a)). TRPV1 levels in ipsilateral L4 and L6 DRGs in SNL group were significantly increased as compared to that in normal group (*P* < 0.05, *P* < 0.05, resp.). EA treatment with 2 Hz frequency fully inhibited SNL-induced increases of TRPV1 protein in ipsilateral L4 and L6 DRGs (*P* < 0.001, *P* < 0.01, resp., compared to SNL group) and even lower than normal TRPV1 levels (*P* < 0.01, *P* < 0.05, resp., compared to normal group; Figures 3(b) and 3(c)). No significant difference of TRPV1 levels in contralateral L5 DRG was observed in rats among normal, SNL, and SNL + EA groups (Figure 3(d)).

### 3.3. EA Inhibited Ipsilateral Undamaged DRG CGRP Upregulation Resulted from SNL

**Figure 4:**
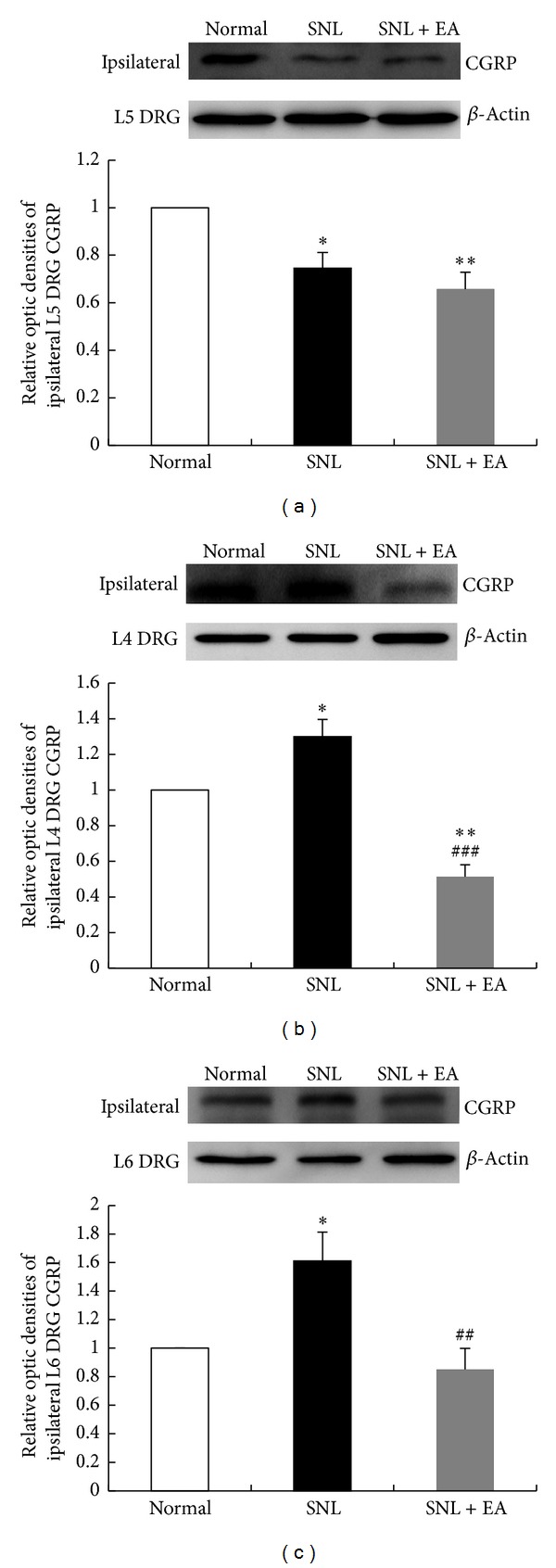
Western blotting analysis of CGRP levels in ipsilateral L5, L4, and L6 DRGs in normal, SNL, and SNL + EA groups. Results were expressed as relative fold changes as compared to normal group after normalization to *β*-actin. Data are presented as mean ± SEM of three independent experiments. *P < 0.05, **P < 0.01, SNL group or SNL + EA group versus normal group; ^##^ *P* < 0.01, ^###^ *P* < 0.001, SNL + EA group versus SNL group.

Since TRPV1 activation promotes CGRP release, which further strengthens TRPV1 role in pain sensation, we also performed western blotting analysis to detect CGRP protein levels. The changes of CGRP levels in ipsilateral DRGs were consistent with the changes of TRPV1 levels. CGRP protein level in ipsilateral L5 DRG in SNL group was significantly decreased as compared to that in normal group (*P* < 0.05), and no significant difference in CGRP protein level was found between SNL and SNL + EA groups (Figure 4(a)). CGRP protein levels in ipsilateral L4 and L6 DRGs in SNL group were significantly increased as compared to that in normal group (*P* < 0.05, *P* < 0.05, resp.), and the increases of CGRP protein in these two DRGs were fully inhibited by 2 Hz EA (*P* < 0.001, *P* < 0.01, resp., compared to SNL group), with even lower expression in L4 DRG in SNL + EA group than that in normal group (*P* < 0.01; Figures 4(b) and 4(c)).

### 3.4. 6′-IRTX Blocked the Antiallodynic Effect of EA

**Figure 5:**
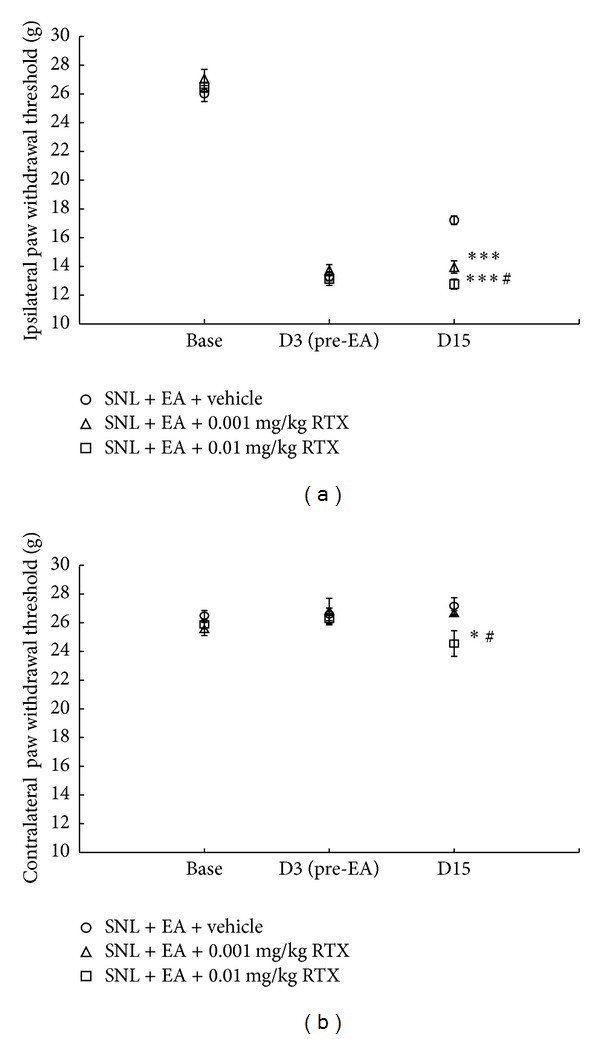
TRPV1 agonist 6′-IRTX blocked the antiallodynic effect of 2 Hz EA. 6′-IRTX was intraperitoneally injected 10 min before 2 Hz EA treatment on day 15. Data are presented as mean ± SEM, *n* = 8 per group. *P < 0.05, ***P < 0.001, SNL + EA + 0.001 mg/kg 6′-IRTX group or SNL + EA + 0.01 mg/kg 6′-IRTX group versus SNL + EA + vehicle group; ^#^ *P* < 0.05, SNL + EA + 0.01 mg/kg 6′-IRTX group versus SNL + EA + 0.001 mg/kg 6′-IRTX group.

To testify EA with 2 Hz frequency alleviated SNL-induced mechanical allodynia, at least in part, by inhibiting ipsilateral undamaged and adjacent DRG TRPV1 up-regulation, we performed block study via using TRPV1 ultrapotent agonist 6′-IRTX. Intraperitoneal injection of 0.001 and 0.01 mg/kg 6′-IRTX greatly reduced ipsilateral PWTs in EA-treated SNL rats (*P* < 0.001, *P* < 0.001, resp., compared to EA treatment-controlled group; Figure 5(a)). The two doses of injection blocked the antiallodynic effect of 2 Hz EA in ipsilateral hind paw, and 0.01 mg/kg 6′-IRTX was more potent than 0.001 mg/kg 6′-IRTX in counteracting 2 Hz EA action (*P* < 0.05). 0.001 mg/kg 6′-IRTX did not significantly change contralateral PWLs of SNL rats that received EA treatment, while 0.01 mg/kg 6′-IRTX significantly reduced contralateral PWL as compared to SNL + EA + vehicle and SNL + EA + 0.001 mg/kg 6′-IRTX groups (*P* < 0.05, *P* < 0.05, resp., Figure 5(b)).

## 4. Discussion

In the current study, the correlation of DRG TRPV1 with low frequency EA analgesia was for the first time investigated in rat neuropathic pain model induced by L5 SNL of the right side. The rats' ipsilateral but not contralateral PWTs were decreased after SNL surgery. It was interesting to find that L5 SNL caused reductions of TRPV1 and CGRP in ipsilateral L5 DRG, but up-regulations in ipsilateral undamaged L4 and L6 DRGs. Low frequency EA alleviated mechanical allodynia and inhibited TRPV1 and CGRP upregulations. Furthermore, TRPV1 agonist 6′-IRTX fully blocked the antiallodynic effect of low frequency EA in ipsilateral hind paw.

Neuropathic pain may arise from nerve compression and trauma, diabetic neuropathy, chemotherapy-caused peripheral neuropathy, spinal cord injury stroke, and viral infections. Despite obvious differences in etiology, many of these pain conditions share common clinical phenomena: spontaneous pain together with allodynia and hyperalgisea [26, 27]. Nerve injury caused by SNL is a classic method to study neuropathic pain [28]. In the current study, rats subjected to L5 SNL developed prominent mechanical allodynia in ipsilateral hind paw. The signs of spontaneous pain such as guarding, licking, and lifting of ipsilateral hind paw were also observed. These results showed that neuropathic pain model was successfully made. Considering that mechanical allodynia was not further serious on day 3 after SNL surgery, we administered 2 Hz EA from day 3 and found that it effectively alleviated mechanical allodynia. Neuropathic pain relief by EA may be frequency specific. A former study showed that 2 Hz EA significantly attenuated hyperalgisea, whereas 120 Hz EA did not exert beneficial effect in diabetic neuropathic pain model [16]. The effectiveness of low frequency EA is also documented in other neuropathic pain models [17–19]. 

Changes in phenotype of primary sensory neurons following peripheral nerve injury contribute to allodynia and hyperalgisea in neuropathic pain [29]. TRPV1 is a member of TRP family channels which are ion channels that respond to mechanical, thermal, chemical, and many other stimuli coming from the extra- and intracellular milieu [30]. Activation of TRPV1 in sensory neurons results in pain perception. A former study showed that the enhanced TRPV1 function in injured sensory neurons by SNL was responsible for neuropathic pain [7]. In the present study, it was interesting to find that L5 SNL caused mechanical allodynia and resulted in an increase TRPV1 in the ipsilateral uninjured L4 and L6 DRGs but not in L5 DRG. It suggested that TRPV1 up-regulation in remaining adjacent undamaged sensory neurons may be also crucial to neuropathic pain. However, the reason why TRPV1 up-regulation could not be observed in injured sensory neurons by SNL needs to be further studied. The enhanced TRPV1 in ipsilateral L4 and L6 DRGs was fully inhibited by 2 Hz EA. Former studies also showed that analgesia produced by EA with low frequency was accompanied by inhibition of TRPV1 up-regulation in sensory neurons in diabetic neuropathic pain, nerve growth factor-induced hyperalgisea, and cancer-induced pain [15, 20, 31]. These findings suggest that inhibition of TRPV1 up-regulation in ipsilateral adjacent undamaged DRGs may be involved in alleviation of mechanical allodynia produced by 2 Hz EA. 

Furthermore, we also carried out block study of 2 Hz EA analgesia by using TRPV1 ultrapotent agonist 6′-IRTX. TRPV1 agonist such as capsaicin can not only activate TRPV1 resulting in pain but also desensitize TRPV1 in a large dose leading to analgesia [32, 33]. A former study showed that systemic administration of RTX (0.1 mg/kg, i.p.) could lead to desensitization of TRPV1 resulting in abolishment of SNL-induced hyperalgisea in rats [33]. Thus, 6′-IRTX in smaller doses of 0.01 and 0.001 mg/kg were administered here to abrogate EA analgesia. A single intraperitoneal injection of 0.001 and 0.01 mg/kg 6′-IRTX on day 15 fully blocked the antiallodynic effect of 2 Hz EA in ipsilateral hind paw, which further supported the speculation mentioned above that 2 Hz EA alleviated neuropathic pain induced by SNL, at least in part, through inhibiting TRPV1 up-regulation.

Activation of TRPV1 can lead to the efferent release of proinflammatory neuropeptides [34]. CGRP is a pro-inflammatory neuropeptide implicated in a variety of painful conditions [35–37]. TRPV1-mediated release of CGRP contributes to TRPV1 role in pain sensation [10, 34]. We found that the variation of CGRP caused by SNL was consistent with that of TRPV1, with an increase in the adjacent intact L4 and L6 DRGs and a decrease in the injured L5 DRG. These results indicated interplay between TRPV1 and CGRP, which may further aggravate SNL-induced neuropathic pain. The increased CGRP was also inhibited by 2 Hz EA, which may result from the suppression of the increased TRPV1 and contribute to EA analgesia.

In conclusion, our data indicate that compensatory TRPV1 up-regulation in ipsilateral adjacent undamaged DRGs, with accompanied increased CGRP, may contribute to SNL-induced mechanical allodynia. EA with low frequency may alleviate SNL-induced mechanical allodynia through inhibiting TRPV1 up-regulation. Our study shows that EA with low frequency may be a useful approach for the management of neuropathic pain.
